# Transcriptome Analysis in Chinese Cabbage (*Brassica rapa* ssp. pekinensis) Provides the Role of Glucosinolate Metabolism in Response to Drought Stress

**DOI:** 10.3390/molecules23051186

**Published:** 2018-05-15

**Authors:** Seung Hee Eom, Seung-A Baek, Jae Kwang Kim, Tae Kyung Hyun

**Affiliations:** 1 Department of Industrial Plant Science and Technology, College of Agricultural, Life and Environmental Sciences, Chungbuk National University, Cheongju 28644, Korea; eom0214@naver.com; 2 Division of Life Sciences and Convergence Research Center for Insect Vectors, Incheon National University, Incheon 22012, Korea; bsa1103@inu.ac.kr (S.-AB.); kjkpj@inu.ac.kr (J.K.K.)

**Keywords:** Chinese cabbage, drought stress, differentially expressed genes, bZIP transcription factor, glucosinolate

## Abstract

Although drought stress is one of the most limiting factors in growth and production of Chinese cabbage (*Brassica rapa* L. ssp. pekinensis), the underlying biochemical and molecular causes are poorly understood. In the present study, to address the mechanisms underlying the drought responses, we analyzed the transcriptome profile of Chinese cabbage grown under drought conditions. Drought stress transcriptionally activated several transcription factor genes, including *AP2*/*ERFs*, *bHLHs*, *NACs* and *bZIPs*, and was found to possibly result in transcriptional variation in genes involved in organic substance metabolic processes. In addition, comparative expression analysis of selected *BrbZIPs* under different stress conditions suggested that drought-induced BrbZIPs are important for improving drought tolerance. Further, drought stress in Chinese cabbage caused differential acclimation responses in glucosinolate metabolism in leaves and roots. Analysis of stomatal aperture indicated that drought-induced accumulation of glucosinolates in leaves directly or indirectly controlled stomatal closure to prevent water loss, suggesting that organ-specific responses are essential for plant survival under drought stress condition. Taken together, our results provide information important for further studies on molecular mechanisms of drought tolerance in Chinese cabbage.

## 1. Introduction

Because the increasing world population and worldwide climate change affects agriculture in several ways, an intergovernmental panel on climate change has concluded that increased concentrations of greenhouse gases will lead to dry conditions in the subtropics, creating widespread drought stress in agricultural regions [1]. Drought is considered as the dominant factor reducing crop growth and yield, and is expected to cause serious problems in plant growth and crop production over more than 50% of all agricultural lands by 2050 [2]. In addition, it has been shown that drought sensitivity in crops has increased over the past twenty years [3]. Therefore, improving drought tolerance is a major goal for crop breeders. Under drought conditions, plants display various physiological and biochemical responses at the cellular and whole-plant levels, leading to a range of specific and nonspecific phenotypes [4]. These responses include stomatal movement, repression of cell growth, development, and photosynthesis, and alteration in biosynthetic pathways, antioxidant pathways, and the respiration pathway [5], suggesting that drought tolerance is an outcome of a series of molecular, cellular, physiological, and biochemical processes mediated via induction and/or repression of genes and their regulation through complex transcriptional networks [6]. This indicates that particular attention to drought-stress response genes and drought-stress-induced transcriptional networks will be required for successful yield protection against drought.

Chinese cabbage (*Brassica rapa* L. ssp. pekinensis) is a subspecies of *B. rapa* and is considered an economically important cruciferous vegetable in Asia, particularly in China, Korea, and Japan. Given its significant economic value and its genetically close relationship with *Arabidopsis thaliana*, Chinese cabbage has been used as a model crop for studies on functional genomics of *Brassica* species and the evolution of polyploid genomes [7]. In addition, high-throughput techniques have been widely used for understanding molecular mechanisms in Chinese cabbage in response to environmental stresses. For example, transcriptome analysis using digital gene expression profiling suggested that genes encoding transcription factors (TFs) including NAC, MYB, HSF (heat shock factor), WRKY, bHLH (basic helix-loop-helix), and ERF (ethylene-responsive factors), antioxidant proteins (superoxide dismutase, peroxidase, catalase, and glutathione S-transferase), and proteins involved in osmolyte synthesis contribute to salt tolerance [8]. Further, comparative transcriptome analysis of different varieties of Chinese cabbage has revealed common and variety-specific responsive transcripts, which can serve as a helpful resource to explore novel candidate genes for improving stress tolerance [9]. Although further functional characterization of these genes will be essential to address how they modulate stress-tolerance, analysis of transcriptome changes help in understanding the molecular basis of plant adaptation to environmental stresses. Morphological, physiological, and biochemical changes in Chinese cabbage under drought conditions have been well-defined and extensively studied [10,11,12], yet little is known about the genome-wide responses of transcripts to drought stress.

Recently, with the development of next-generation sequencing (NGS) technology, RNA-sequencing (RNA-seq) for transcriptome analysis has been widely used to identify differentially expressed genes (DEGs) among different treatment, tissues or growth periods, suggesting that RNA-seq is powerful tool to obtain an overall view of gene expression profiles [13]. In the present study, to investigate the molecular basis of response to drought stress in Chinese cabbage, the transcriptomes of leaves and roots under drought conditions were analyzed using digital gene expression profiling. Drought stress-inducible or -repressible genes were identified, and further classified as common or specifically regulated. In addition, comparative analysis of DEGs in leaves and roots revealed the importance of glucosinolate metabolism in controlling the response to drought conditions. Taken together, our results provide an overview of molecular mechanisms triggered by drought stress in plants, and will be helpful in unraveling the basic mechanisms of environmental stress tolerance.

## 2. Results and Discussion

### 2.1. Physiological Response to Drought Stress in Chinese Cabbage

**Figure 1:**
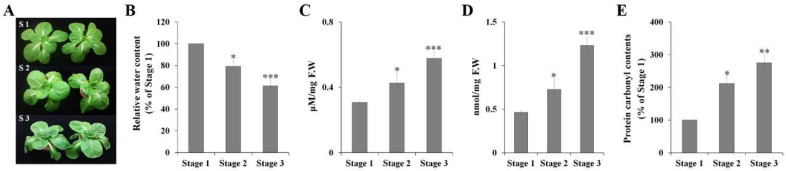
Physiological response to drought stress in Chinese cabbage. (**A**) Phenotypes of Chinese cabbage plants after exposure to drought stress for four days. S1 (Stage 1), normal water supply; S2 (Stage 2), water withholding for 2 d (soil water content at 20%); S3 (Stage 3), water withholding for 4 d (soil water content at 5%). Relative water content (**B**) and changes in levels of H_2_O_2_ (**C**), MDA (**D**), and protein carbonylation (**E**) after drought treatment were determined. Values are averages from three biological replicates consisting three plants per sample were used. The data are presented as mean ± standard error (SE). * *p* < 0.05, ** *p* < 0.01, and *** *p* < 0.001 represent the significant differences in comparison with Stage 1 (S1).

Drought stress is a major abiotic stress, inducing accelerated production of several reactive oxygen species including superoxide, singlet oxygen, hydroxyl radicals, and H_2_O_2_ causing oxidative damage to proteins, DNA, and lipids in different cellular compartments [14]. Therefore, oxidative stress markers including lipid peroxidation in terms of MDA, ROS accumulation, and protein carbonylation have been analyzed to identify variations in physiological response to drought stress [15,16,17]. We analyzed the physiological responses of Chinese cabbage subjected to drought stress, to evaluate efficacy of the treatment. As shown in Figure 1B, drought stress induced significant changes in relative water content. The leaves of the plant showed accumulation of H_2_O_2_ (Figure 1C), MDA (Figure 1D), and protein carbonylation (Figure 1E), with their levels increasing 1.9-, 2.7-, and 2.7-fold respectively after four days of drought stress treatment (Stage 3), compared with those in the control plants (Stage 1) (Figure 1A). These physiological changes suggested that the drought stress treatment was effective, and the leaves and roots of Chinese cabbage plants grown under control conditions (Stage 1) and drought stress conditions (Stage 2 and Stage 3) were harvested to unravel the molecular basis of the response.

### 2.2. RNA-Seq and Identification of Drought-Responsive Genes

During the last decade, several exciting studies have reported the development of new disease-resistant crops using genetic engineering tools, but efforts to generate drought-tolerant crops has been less successful [18]. This might be due to the complex responses of crops to drought stress. In fact, drought stress induces various morphological, physiological, and biochemical changes in crops controlled by numerous small-effect loci and hundreds of genes [19], indicating that knowledge of the biochemical and molecular responses to drought should contribute to improving plant tolerance under water-limited conditions. Therefore, to obtain a global overview of molecular mechanisms involved in drought stress response, total RNA was extracted from roots and leaves of control and drought-treated plants and used for generation of transcriptome libraries on an Illumina HiSeq™ 2500 sequencing platform. After removing low-quality reads, 46 to 54 million clear reads (4.64 to 5.49 Gb) from each sample were acquired for further analysis (Table 1).

**Figure 2:**
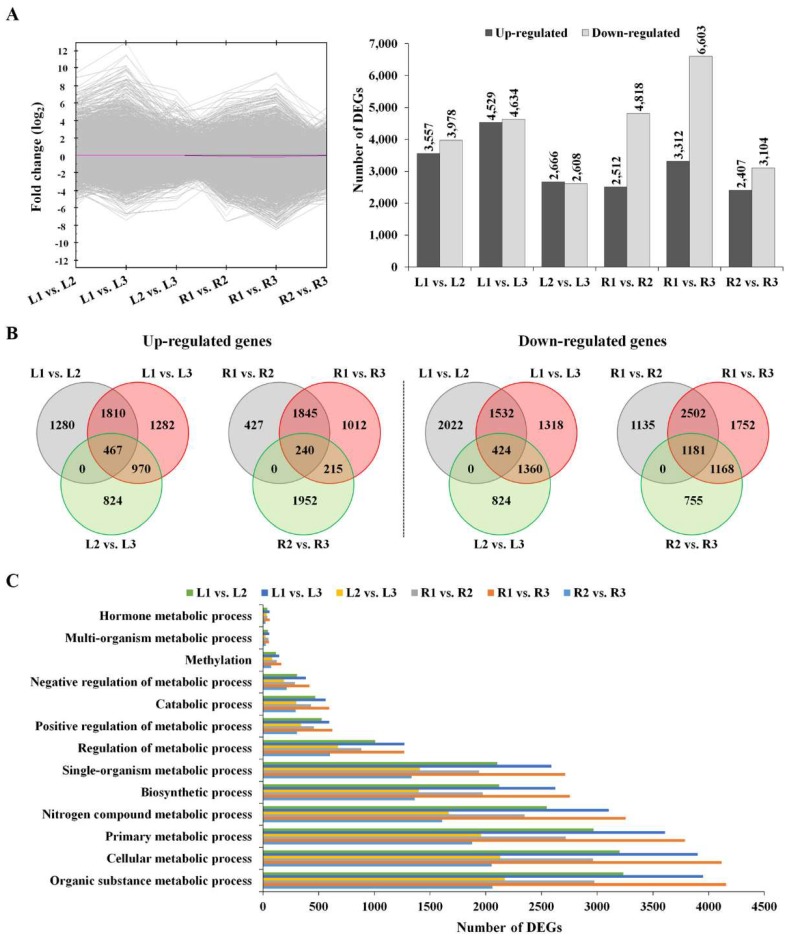
Analysis of differentially expressed genes (DEGs). (**A**) Distribution of up- and down-regulated DEGs in each comparison; (**B**) Venn diagram analysis of DEGs in different comparisons among groups; (**C**) Classification of DEGs based on metabolism categories. L1, L2, and L3, leaf samples obtained from Stage 1, Stage 2, and Stage 3 plants, respectively; R1, R2, and R3, root samples obtained from Stage 1, Stage 2, and Stage 3 plants, respectively.

All reads were deposited in the National Agricultural Biotechnology Information Center (NABIC, http://nabic.rda.go.kr). To identify the DEGs in the control and drought-treated groups, we constructed six comparison groups, and DEGs were defined as genes with value of |log2 (fold change)| ≥ 1. As shown in Figure 2A, a total of 7535 DEGs (3557 up-regulated and 3978 down-regulated) were detected comparing the L1 and L2 libraries. Comparative analysis of the L2 and L3 libraries showed that the expression of 2666 genes was increased and that of 2608 genes was down-regulated in L3. Upon drought stress exposure, more DEGs were down-regulated in roots than in leaves (Figure 2A), indicating that the response mechanism against drought stress differs between roots and leaves. A total of 1280 genes were exclusively up-regulated in L2, and the expression levels of 1282 genes were increased in L3 but not in L2. In roots, transcripts of 1135 genes were down-regulated solely in R2, whereas 1752 transcripts were down-regulated exclusively in R3 but not in R2 (Figure 2B). Further, 467 DEGs from leaves and 240 DEGs from roots were found to be up-regulated in both tissues, whereas 424 DEGs from leaves and 1181 DEGs from roots were found to be down-regulated in both tissues (Figure 2B). *Non-specific lipid-transfer protein 3* (XP_009132002.1), *dehydrin Rab18-like* (XP_013609020.1), and *sucrose synthase 3* (XP_009111303.1) were among those identified to be up-regulated in both roots and leaves, whereas *aquaporin TIP2-1* (XP_013653350.1) was down-regulated in both tissues.

To understand the universal response of Chinese cabbage to drought stress, all screened DEGs in the six comparison groups were subjected to GO enrichment analysis, and 60 functional groups classified into three categories comprising “cellular component,” “molecular function,” and “biological process” were identified (Table S1). The category “biological process,” consisting of 27 functional groups, exhibited the highest number of annotations alongside “cellular process”, followed by “metabolic process”, and “single-organism process”. In addition, the drought-induced DEGs revealed various affected metabolic processes, mainly the organic substance metabolic processes, cellular metabolic processes and primary metabolic processes (Figure 2C). Photosynthesis is one of the important processes affected by drought, due to changes including decrease in turgor pressure, reduction in CO_2_ assimilation and diffusion, and impairment in the photosynthetic apparatus [20,21,22], suggesting that alterations in primary metabolic processes under drought conditions (Figure 2C) are mediated via drought-induced photosynthesis inhibition. 

Further, transcript abundances of genes involved in the abscisic acid (ABA) biosynthesis and degradation pathway were dynamically changed in response to drought stress, whereas a number of genes involved in biosynthesis of auxin, gibberellin, or ethylene were down-regulated (Figure S1), suggesting that the hormonal regulation of drought stress response in Chinese cabbage is primarily mediated via ABA.

### 2.3. Transcription Factors and MapMan Analysis of Chinese Cabbage Genes Associated with Drought-Stress Response

Transcription factors (TFs) are known as major players in various transcriptional regulatory mechanisms in stress-induced signal transduction pathways. Therefore, understanding the role of stress-inducible TFs is important for engineering stress-tolerant plants by modulating a large set of genes [23]. In Chinese cabbage transcriptome libraries generated in the above mentioned RNA-seq, a total of 3663 TFs mainly distributed into 45 TF families was found (Figure S2). Among these TFs, the most abundant differentially expressed TF family was the AP2/ERF family (Figure S3). Based on the difference in copy numbers and similarity of AP2/ERF domains, this family is further divided into five subfamilies, the Apetala2 (AP2), the ethylene responsive element binding factor (ERF), the Related to ABI3/VP1 (RAV), the dehydration-responsive element-binding (DREB) protein, and the Soloist [24], However, our transcriptome libraries did not contain members of the Soloist subfamily (Figure S4), probably due to its tissue-specific expression. In fact, expression of the *Soloist* gene was detected only in the bud of Chinese cabbage plants [25]. The ERF subfamily, which plays significant roles in regulating gene expression in response to biotic and abiotic stresses [6], was the most abundant class of the AP2/ERF family differentially expressed in response to drought (Figure S4).

**Figure 3:**
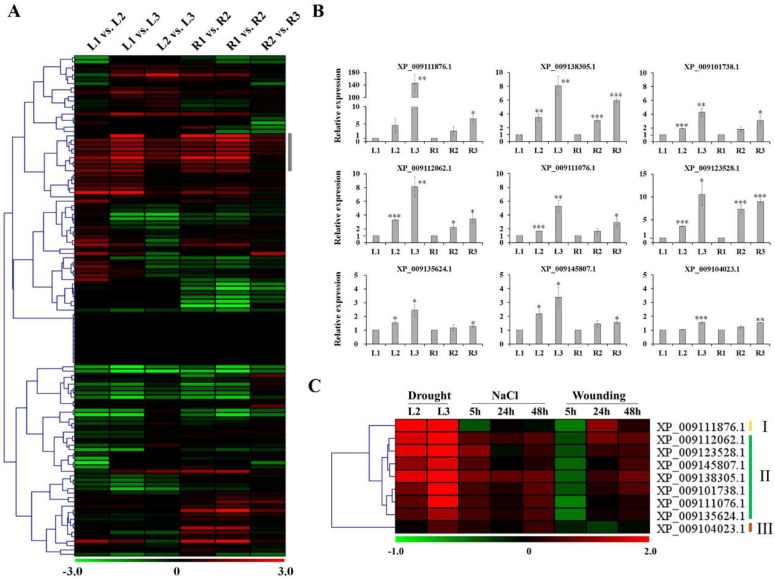
Change in bZIP transcription factor (TF) expression during the response to abiotic stresses. (**A**) The heatmap shows the expression profiles of bZIP TFs in different comparisons among groups. The grey bar indicates bZIP TFs selected for qRT-PCR analysis; (**B**) The expression patterns of the selected bZIP TFs were analyzed using qRT-PCR. Transcript levels of the selected bZIP TFs were normalized to those of Chinese cabbage actin, and gene expression is relative to Stage 1 plants (L1 or R1) set to a value of 1 for each biological replicate. Values are averages from three independent biological experiments. Data are means ± SE. * *p* < 0.05, ** *p* < 0.01, and *** *p* < 0.001 represent the significant differences in comparison with L1 or R1; (**C**) Differential expression of the selected bZIP TFs under different stress treatments. Expression is indicated as a log2 ratio of experimental treatments relative to control samples and visualized in heatmaps showing hierarchical clustering of the selected bZIP TFs.

The bZIP TF family, which contain a basic region for DNA-binding and a leucine zipper motif for dimerization, is one of the largest and most diverse TF families in plants [26], and plays diverse functions in plant growth and development and environmental stress responses, especially in ABA signaling related to stress responses [27]. Out of 136 bZIP TFs identified in *B. rapa* (BrbZIP) [28], 132 bZIP TFs were found in Chinese cabbage transcriptome libraries, and exhibited differential expression patterns (Figure 3A). Among them, 64 bZIP TFs were mainly differentially expressed in drought-treated plants. A total of 17 bZIP TFs were increased in both leaves and roots of drought-treated plants, and five bZIP TFs were only up-regulated in leaves. In addition, seven bZIP TFs were only down-regulated in leaves, six bZIP TFs only in roots, and three bZIP TFs were down-regulated in both roots and leaves. Drought stress-responsive gene expression is regulated by TFs operating in ABA-dependent and ABA-independent signaling pathways. Among these TFs, members of the bZIP TF family regulate the transcriptional induction of genes directly or indirectly linked with stress tolerance in plants [29]. In Arabidopsis, accumulated evidence has suggested that group-A bZIP including AtbZIP35, AtbZIP36, AtbZIP37, AtbZIP38, AtbZIP39, AtbZIP40, and AtbZIP66 play crucial roles in activating plant ABA signaling or in abiotic stress response [30,31]. We also found that *BrbZIP23* (XP_009112062.1), *BrbZIP15* (XP_009101738.1), *BrbZIP17* (XP_009123528.1), *BrbZIP21* (XP_009135624.1), *BrbZIP22* (XP_009145807.1), and *BrbZIP10* (XP_009104023.1), belonging to group-A BrbZIPs, were involved in drought response (Figure 3B). In addition, *BrbZIP101* (XP_009111876.1), *BrbZIP114* (XP_009138305.1), and *BrbZIP115* (XP_009111076.1), belonging to group-S bZIPs, were also found to be induced under drought conditions. Although the physiological function of group-S bZIPs remains poorly understood, the increased transcription levels of group-S bZIPs due to abiotic stresses might suggest their regulatory roles in stress response in plants [32,33,34,35]. Further, poplar *PtabZIP1-like* (*PtabZIP1L*), homologous to the *Arabidopsis bZIP1* belonging to group-S *bZIPs*, has been known to be a positive modulator of drought resistance acting through modulation of multiple metabolic pathways including flavonoid biosynthesis [36]. These findings indicate that drought-induced group-S *BrbZIPs* (Figure 3C) might be interesting candidate genes for improving drought tolerance.

To validate the gene expression results from RNA-seq, the expression of nine bZIP TFs up-regulated in both roots and leaves was analyzed using qRT-PCR. As shown in Figure 3B, the expression pattern of these genes was found to vary in response to drought. In addition, exposure of Chinese cabbage to NaCl or wounding also significantly induced or repressed the expression of these genes, although the changes were relatively lower than those due to drought treatment (Figure S5). Further, selected bZIP TFs were found to show complex expression patterns in response to drought, NaCl, and wounding, as evidenced by cluster analysis results represented as a heatmap (Figure 3C). In cluster I, *BrbZIP* (XP_009111876.1), also known as *BrbZIP101* [26] exhibited relative higher transcript accumulation in drought-treated leaves and was transiently down-regulated in response to NaCl and wounding stresses, whereas *BrbZIPs* in cluster II only showed transient down-regulation due to wounding stress. These results indicate that *BrbZIPs* have divergent functions in response to environmental stresses, and the increased expression of *BrbZIP101* might suggest its role as a marker of drought-stress response.

**Figure 4:**
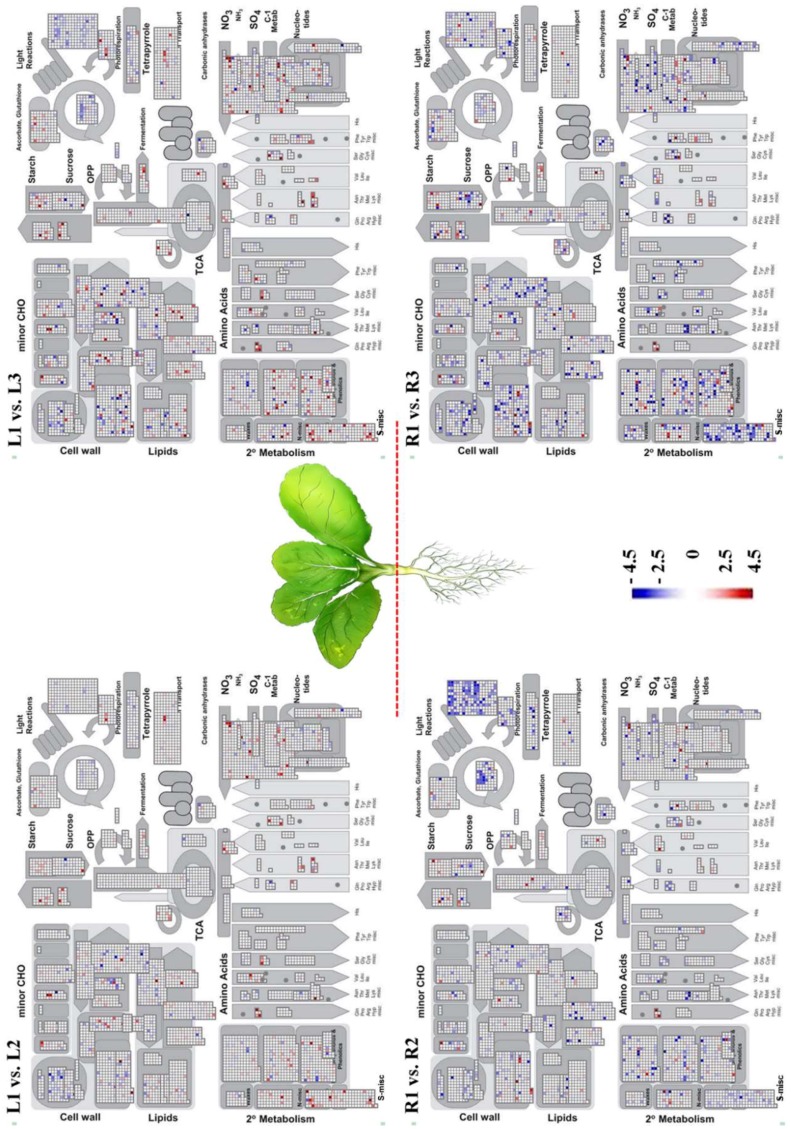
Mapman metabolism overview showing the differentially expressed genes (DEGs) in each comparison. The different colors represent the log2 values of the gene expression levels in response to drought stress.

Functional enrichment analysis with respect to known gene ontologies using MapMan can be performed to obtain a global overview of high-throughput data in the context of metabolic pathways and biological processes [37]. To investigate the metabolic pathways affected by drought stress, MapMan analysis was performed on L2/L1, L3/L1, R2/R1, and R3/R1 comparisons based on DEGs. In leaves and roots of drought-treated plants, several metabolic pathways including those involving cell wall synthesis, light reactions, and the Calvin cycle were found to be down-regulated, whereas those involving fermentation, starch synthesis, and amino acid synthesis were up-regulated (Figure 4). However, the impact of drought stress on the transcript levels of genes involved in these metabolic pathways in roots was higher than that in leaves. Noticeably, the genes involved in the sulfur-containing secondary metabolites (S-misc) category pathways were up-regulated in the leaves but down-regulated in the roots under drought-stress conditions, indicating differences between organs of Chinese cabbage in response to drought stress.

### 2.4. Alteration in Glucosinolate Metabolism in Rresponse to Drought Stress

**Figure 5:**
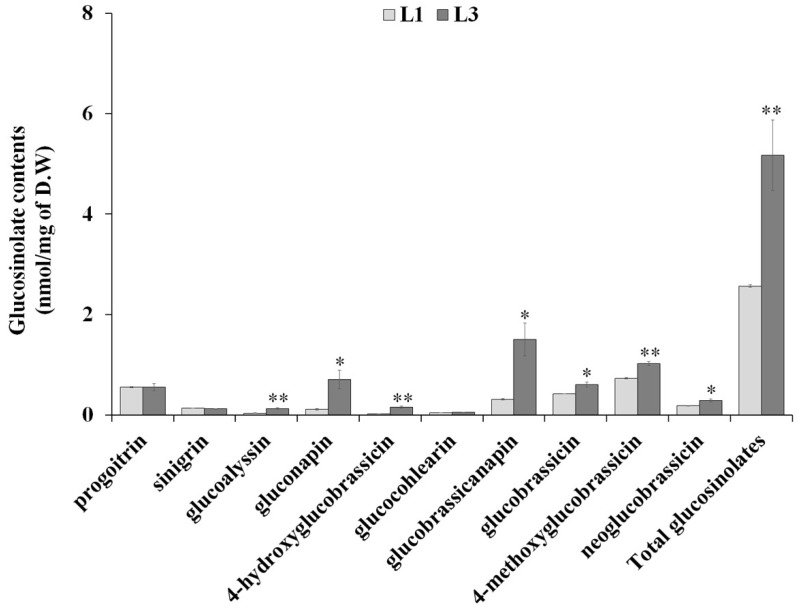
Glucosinolate content in control (L1) and drought-treated (L3) Chinese cabbage leaves. Values are averages from three biological replicates. Error bars indicate S.E. * *p* < 0.05 and ** *p* < 0.01 indicate significantly different from L1.

In higher plants, a variety of sulfur-containing secondary metabolites are synthesized, which often play a crucial role in the survival of plants under biotic and abiotic stresses [38]. The alteration in sulfur metabolism under drought conditions is consistent with the promotion of root growth [39]. The down-regulation of glucosinolate metabolism in roots upon drought suggests a switch in sulfur metabolism from glucosinolate to other metabolic processes, which are required for enhancing root growth. Interestingly, we found that the genes involved in sulfur-containing secondary metabolites have opposing expression patterns in leaves and roots during response to drought stress (Figure 4). With respect to the S-misc BIN category, the genes mainly represented were those responsible for glucosinolate biosynthesis including *branched-chain amino acid aminotransferase 4* (*BCAT4*) and *methylthioalkylmalate synthase 1* (*MAMI1*) (Figure S6, Table S2). To test whether the increased expression of these genes correlated with the accumulation of glucosinolates, we analyzed drought-induced variation in glucosinolate composition and content. As shown in Figure 5, the total glucosinolate content was increased in response to drought stress in leaves of Chinese cabbage, suggesting that the accumulation of glucosinolates in L3 was due to increase transcription of glucosinolate biosynthesis genes.

In addition, variation in the levels of identified glucosinolate compounds between non-stressed plants (L1) and drought-treated plants (L3) was observed. The compound 4-methoxyglucobrassicin (0.73 ± 0.01 nmol/mg of D.W) was found to be the major constituent in Chinese cabbage cultivars (Chunkwang), followed by progoitrin (0.55 ± 0.01 nmol/mg of D.W) and glucobrassicin (0.42 ± 0.001 nmol/mg of D.W), whereas glucobrassicanapin (1.50 ± 0.02 nmol/mg of D.W) and 4-methoxyglucobrassicin (1.02 ± 0.002 nmol/mg of D.W) were the abundant glucosinolates in L3. Further, drought-stress treatment led to an approximately six-fold increase in levels of gluconapin and 4-hydroxyglucobrassicin, indicating that drought stress increased the transcription level of genes involved in the glucosinolate biosynthetic pathway (Figure S6) and glucosinolate accumulation in the leaves of Chinese cabbage (Figure 5), consistent with the protein competition model which predicts the effects of environmental factors on secondary metabolism [40,41]. Similarly, the accumulation of glucosinolates has been observed in *Brassica* species as a part of the plant’s response to drought, although this response was highly variable depending on the duration and intensity of drought [40]. These findings indicate that drought stress significantly influenced the glucosinolate composition and content through modulating the expression of a range of glucosinolate biosynthesis genes.

**Figure 6:**
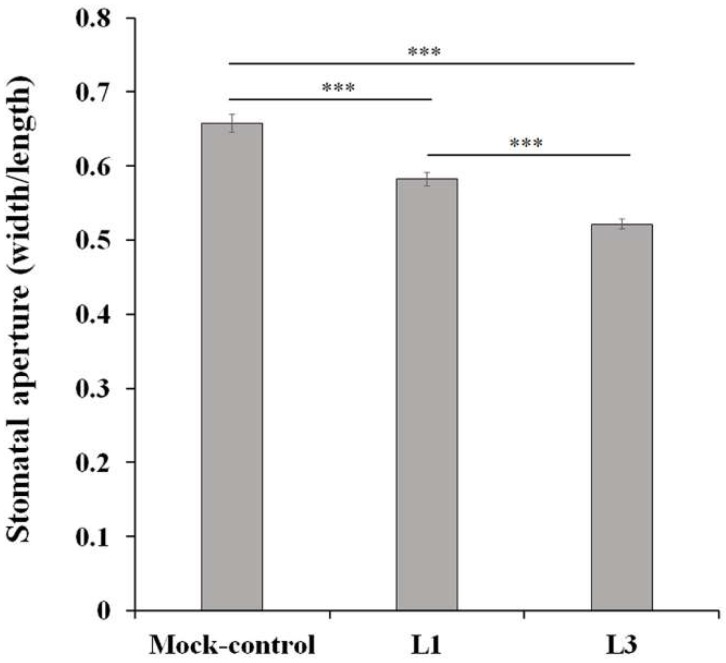
Stomatal movement in Chinese cabbage in response to glucosinolate-derived products obtained from control (L1) and drought-treated (L3) Chinese cabbage leaves. Apertures were analyzed as width/length after 2 h of treatment. Values are means ± S.E., *** *p* < 0.001, *n* > 100.

Glucosinolate degradation products generated by the enzymatic action of myrosinases have attracted attention for several years due to their important role in plant defense against pests and pathogens [42]. In addition, the multiple roles of glucosinolates and their degradation products in heat tolerance, water transport, and transcriptional reprogramming suggest the physiological importance of these compounds in plant response to environmental stresses [43,44,45]. Exogenous application of glucosinolate degradation products, isothiocyanates, nitriles, and thiocyanates has been found to induce stomatal closure, allowing suppression of water loss [46,47]. Here, we hypothesized that drought-induced accumulation of glucosinolates may regulate stomatal closure leading to suppression of water loss. To test this hypothesis, glucosinolate-derived products were extracted from L1 and L3 samples and were applied to leaf disks floated on the opening medium. Application of glucosinolate-derived products significantly reduced stomatal apertures compared with application of mock-control (solvent control) (Figure 6).

The movement of the stomatal guard cells was found to be more responsive to glucosinolate-derived products obtained from L3 than to those obtained from L1 (Figure 6), indicating that the accumulation of glucosinolates induced drought tolerance through stimulating stomatal closure. Taken together, the increased concentration of sulfate transported from the roots to the leaves might be required for enhancing the anti-transpiration effect of ABA in the stomata in leaves [48,49], and glucosinolate degradation products function analogously to ABA in stomatal aperture regulation [50]. These findings suggest that sulfur metabolism plays significant roles in drought stress signaling and responses [51] and indicate that variation in glucosinolate metabolism between roots and leaves (Figure 5) is required for successful defense against drought conditions.

## 3. Materials and Methods

### 3.1. Plant Material and Stress Treatment

Seeds of Chinese cabbage (*B. rapa* L. subsp. pekinensis) cultivar Chunkwang were obtained from the Sakata Korea Seed Co. Ltd. (Seoul, South Korea), and were germinated and grown in a growth chamber with a long photoperiod (16 h light/8 h dark) at 24 °C. Soil water content was measured using HH2 Moisture Meter (Delta-T Devices, Cambridge, UK). The water content in the soil was controlled to be 60 to 65% of saturated soil water content. For drought stress treatment, 4-week-old plants (S1) were subjected to water withholding for 2 d or 4 d, resulting in a soil water content of 20% (S2) or 5% (S3), respectively. In addition, 4-week-old plants were watered with 250 mM NaCl or effectively wounded using sterile syringe needles to analyze the expression pattern of bZIP TFs. Each treatment was carried out three times, resulting in three biological replicates.

### 3.2. Determination of Relative Water Content, Malondialdehyde (MDA) Content, H_2_O_2_ Accumulation, and Protein Carbonyl Content

Relative water content was determined according to Soltys-Kalina et al. [52], and MDA content was determined using the thiobarbituric acid (TBA) reaction as described by Spanic et al. [53]. About 200 mg of ground material obtained from each sample (leaves from S1, S2, and S3) was used. MDA concentration was estimated by subtracting the non-specific turbidity at 600 nm from the absorption at 532 nm, and was expressed as MDA in nmol per mg of fresh weight using an absorbance coefficient of extinction (155 mM^−1^ cm^−1^).

As described by Spanic et al. [53], the supernatant obtained via 0.1% ice-cooled TCA extraction was used to analyze H_2_O_2_ concentration. After reaction with 1 M KI, the absorbance at 390 nm was determined via comparison to a standard curve prepared using H_2_O_2_. H_2_O_2_ concentration in each sample was expressed as µM per mg of fresh weight.

For protein carbonyl determination, 300 mg of frozen dried samples were incubated with protein extraction buffer containing 50 mM Tris-HCl, pH 7.5, 1 mM EDTA, 5 mM MgCl_2_, and 10 mM β-mercaptoethanol. After centrifugation at 12,000 g for 15 min at 4 °C, the protein content in the supernatant was determined following the Bradford method with bovine serum albumin as standard. Protein carbonyl content was determined using a fluorometric protein carbonyl content assay kit (BioVision, Milpitas, CA, USA) following the manufacturer’s instructions. 

### 3.3. Identification and Functional Annotation of DEGs

Total RNA was extracted from each sample using the FavorPrep Plant Total RNA Mini Kit (Favorgen Biotech Corp, Pingtung, Taiwan), and quantified using an DeNovix DS-11 (DeNovix Inc., Wilmington, DE, USA). Equal amounts of total RNA from different stages of each sample were pooled to generate a cDNA library. Equal amounts of total RNA from different stages of each sample were pooled to generate a cDNA library. After DNaseI treatment, poly(A) mRNA was isolated with oligo(dT) magnetic beads and fragmented to short fragments. Then, the cDNA library was synthesized as described by Choi et al. [54] and sequenced using an Illumina HiSeq™ 2500 sequencing platform. The quality of raw data was controlled by using the FastQC tool, and adapter sequences, empty reads, low-quality reads (with ambiguous sequence, N), and reads with more than 10% Q < 20 bases (i.e., with a base quality of less than 20) were discarded using Trimmomatic v.0.33. The clean reads were mapped to the *B. rapa* reference sequence (BrapaV2.1, http://brassicadb.org/brad/datasets/pub/Genomes/Brassica_rapa/V2.0/V2.1/Chr/) using the HISAT2 aligner [55]. Transcript levels were calculated using SAMtools (http://samtools.sourceforge.net/) and relative transcript abundances were analyzed using DEseq [56]. DEGs were determined by combining a *p* value cutoff of 0.01 and adjusting to |log2 (fold change)| ≥ 1.For functional annotation, gene ontology (GO) enrichment analysis of DEGs was performed using Blast2GO (v.4.1.0) In addition, MapMan metabolism overview maps were drawn as described by Yu et al. [57].

### 3.4. Quantitative Real-Time PCR (qRT-PCR) Analysis

To further verify the expression profiles observed in the RNA-Seq data, nine bZIP TFs were selected for qRT-PCR analysis performed using the SYBR^®^ Green Real-time PCR Master Mix (TOYOBO, Co., Ltd., Osaka, Japan) in the CFX96TM Real-time system (Bio-Rad, Hercules, CA, USA) with default parameters. The conditions for all reactions were as follows: 95 °C for 60 s, 40 cycles of 95 °C for 5 s, followed by 60 °C for 15 s, and 72 °C for 20 s. Melting curve analysis was performed to confirm the PCR specificity. The relative expression levels of selected bZIP TFs were normalized to expression of the internal reference gene actin [58]. Specific primer pairs used in qRT-PCR are listed in Table S3.

### 3.5. HPLC Analysis of Glucosinolates

Extraction and desulfation of glucosinolates were performed following the method described by Kim et al. [59]. Desulfated glucosinolates were analyzed using an HPLC system (1200 series, Agilent Technologies, Waldbronn, Germany) with an Inertsil ODS-3 (C18) column (4.6 mm × 250 mm, particle size 5 μm, GL Sciences, Tokyo, Japan). The mobile phase consisted of solvents A (water) and B (acetonitrile) with a gradient elution comprising 0 min (99% A), 0–18 min (1–30% B, linear gradient), and 18–30 min (30% B, isocratic). Glucosinolate content was calculated as sinigrin equivalents [60].

### 3.6. Measurement of Stomatal Aperture

Stomatal apertures were measured as described by Hossain et al. [47]. Briefly, leaf disks were floated on the opening medium (5 mM KCl, 50 μM CaCl_2_ and 10 mM MES-Tris, pH 6.15) for 2 h under light conditions. To analyze the effect of glucosinolate-derived products on stomatal movement, glucosinolate-derived products was extracted from 50 mg of frozen dried samples (leaves from S1 and S3) as described by Lola-Luz et al. [61]. Each extract was re-suspended in 5 mL HPLC-grade methanol and filtered using a membrane filter (0.2 μm pore size). Then, leaf disks floated on the opening medium were treated with 5% of extract (50 μL of each extract suspended in 5 mL MeOH was mixed with 1 mL of opening medium). Two hours after treatment, stomatal apertures were captured using an CKX53 microscope (Olympus, Tokyo, Japan) and the width and length of 100 stomatal apertures were measured using the image processing software ImageJ (National Institute of Mental Health, Bethesda, MD, USA).

## 4. Conclusions

Because of the complexity of drought as a stress signal, understanding the biochemical and molecular mechanisms of plant response has remained a major challenge in plant biology and breeding. The present study provides an overview of the changes in transcript abundance in leaves and roots under drought conditions. DEGs found to be responsive to drought suggest differential regulation patterns in the leaf and root, and drought-induced group-S BrbZIPs are interesting candidates for improving plant tolerance. Further, we suggest that organ-specific metabolic responses to drought are part of the survival strategies of plants. Taken together, our dataset provides a crucial starting point for future efforts in understanding the biochemical and molecular mechanisms underlying response to drought stress in Chinese cabbage.

## Figures and Tables

**Table 1:** Summary of RNA sequencing data from six RNA libraries of control and drought-stressed leaves and roots.

Sample No.	Tissue	Drought Stress	Clean Reads	Clean Bases (Gb)	Accession Number (NABIC)
L1	Leaf	Stage 1	53,744,064	5.38	NN-4956
L2	Leaf	Stage 2	46,299,312	4.64	NN-4958
L3	Leaf	Stage 3	54,348,542	5.45	NN-4960
R1	Root	Stage 1	54,764,100	5.49	NN-4957
R2	Root	Stage 2	48,558,468	4.87	NN-4959
R3	Root	Stage 3	47,117,410	4.72	NN-4961

## Supplementary Materials

The following are available online.
