# Supplementary material for: Transcriptome Analysis in Chinese Cabbage (Brassica rapa ssp. pekinensis) Provides the Role of Glucosinolate Metabolism in Response to Drought Stress
Source: Molecules. 2018 May 15;23(5):1186. doi: 10.3390/molecules23051186 (PMC6099646; doi:10.3390/molecules23051186)
Supplement: Supplementary file 1 [file molecules-23-01186-s001.pdf]

Supplementary materials

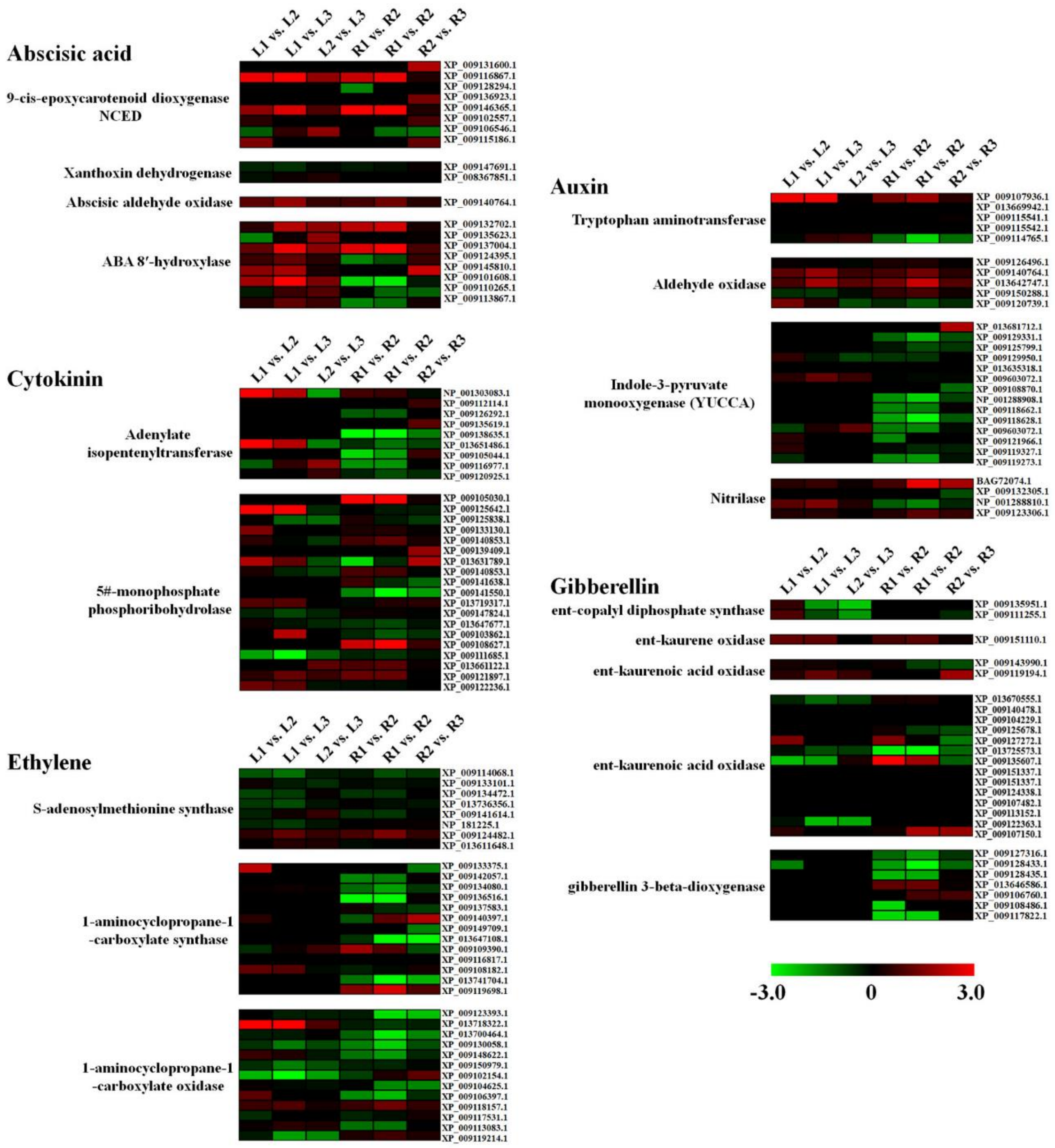

Figure S1. Expression pattern of genes involved in each hormone biosynthesis pathway. The different colors represent the log2 values of the relative expression levels relative to control samples.

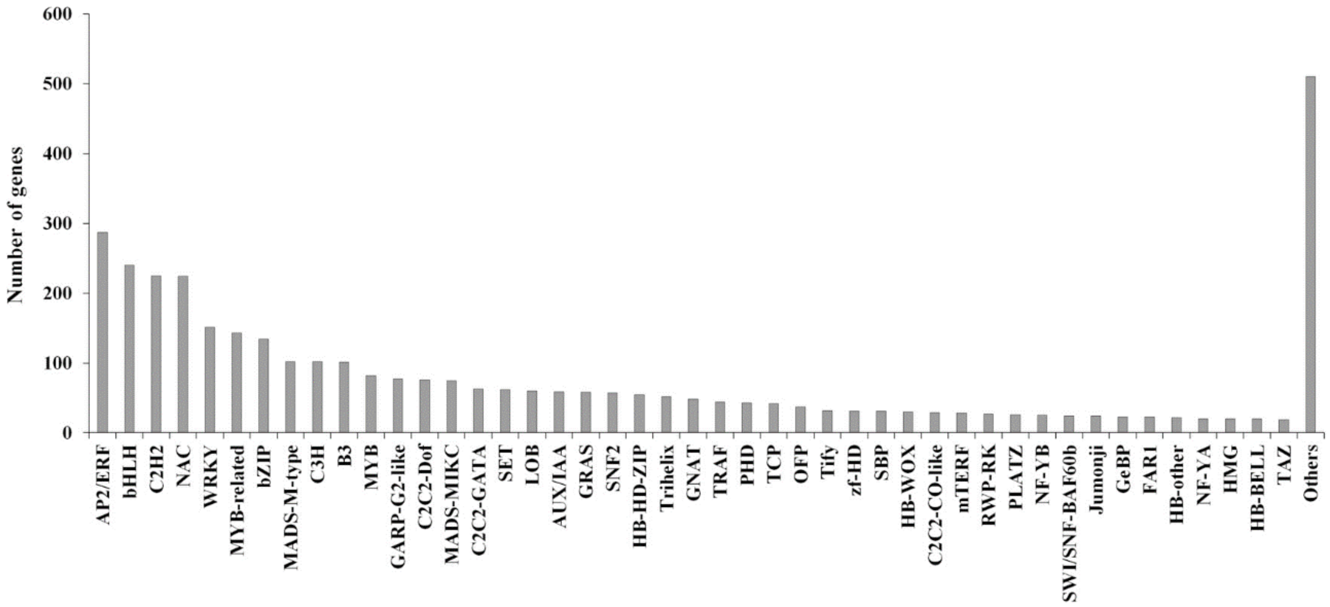

Figure S2. Classification of transcription factors found in Chinese cabbage transcriptome libraries.

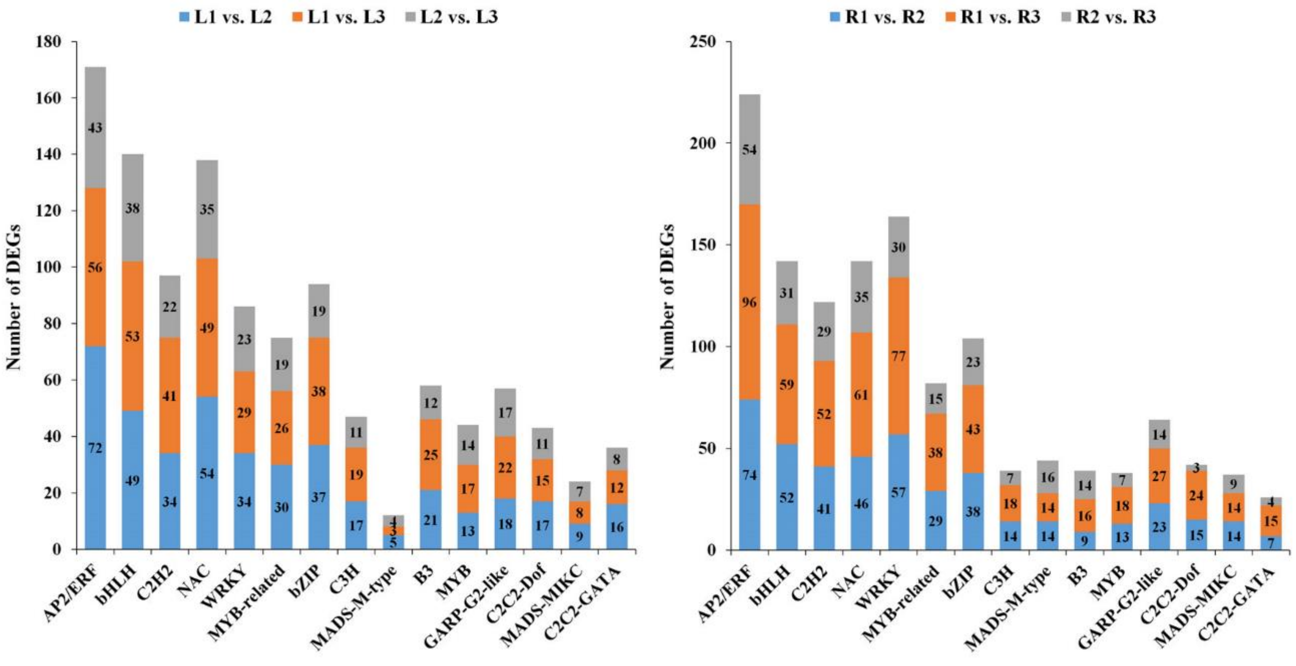

Figure S3. The number of differentially expressed genes (DEGs) identified as transcription factors.

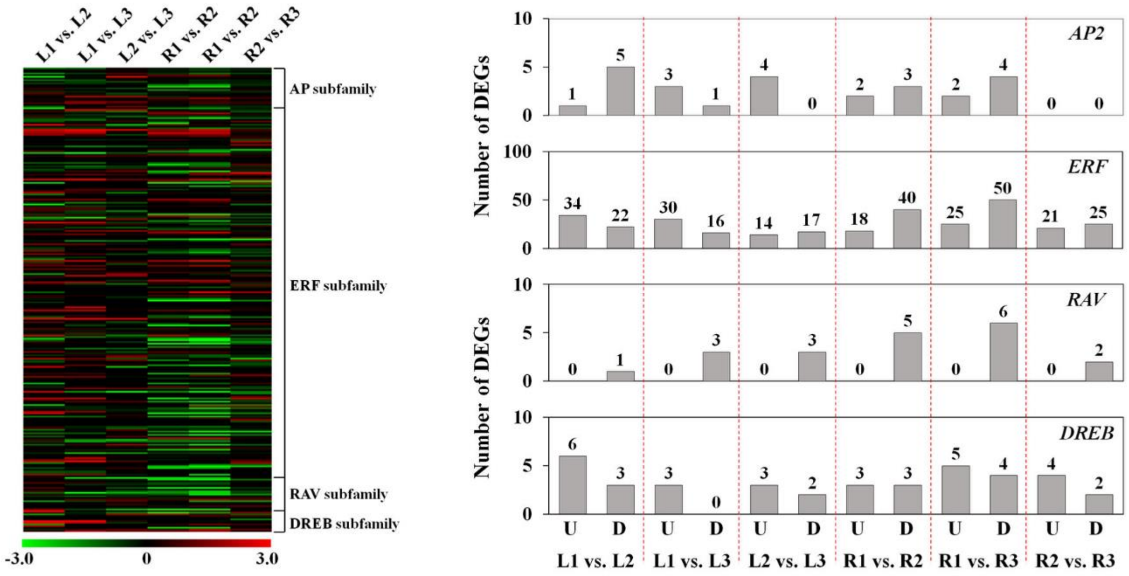

Figure S4. The expression profiles of the AP2/ERF family in different comparisons among groups. U and D indicate up- and down-regulated genes, respectively.

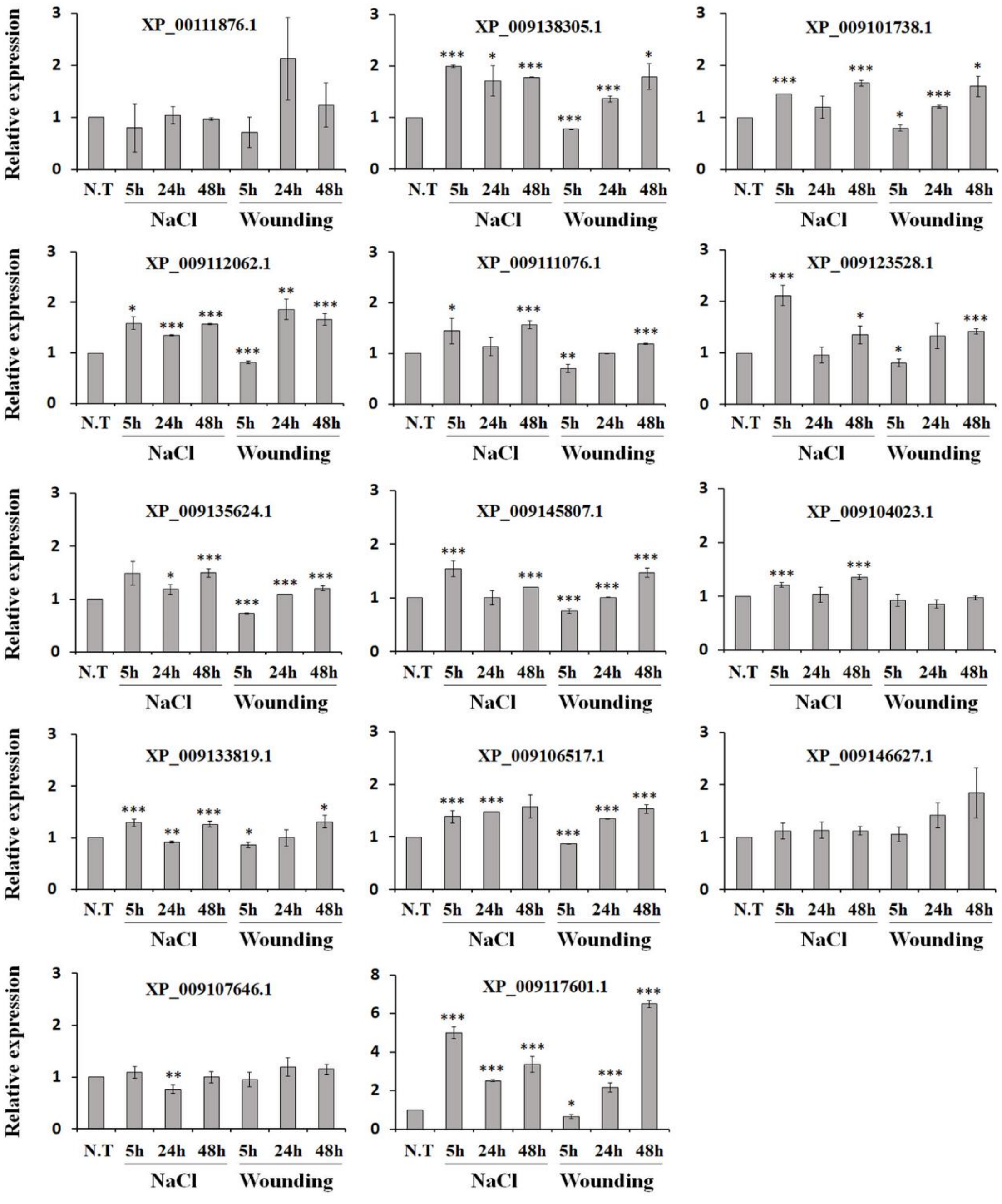

Figure S5. The expression patterns of the selected bZIP TFs in leaves exposed to salt and wounding stresses. Transcript levels of the selected *bZIP* TFs were normalized to that of actin and were expressed relative to the values of N.T (non-treated samples). Values are means ± S.E., * $p < 0.05$, ** $p < 0.01$, and *** $p < 0.001$ represent the significant differences in comparison with N.T.

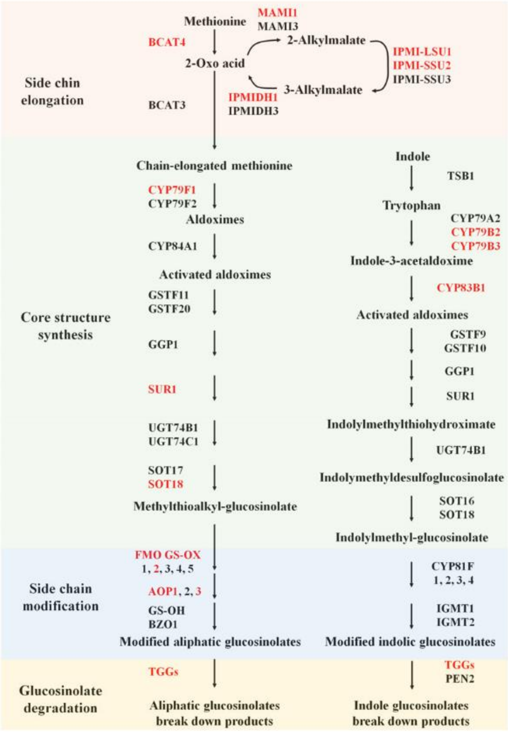

		Gene	Gene bank ID	Log2 Fold Change			
				L1 vs. L2	L1 vs. L3	R1 vs. R2	R1 vs. R3
Aliphatic pathway	Side chain elongation	BCAT4	XP_009135648.1	2.4936	2.5337	-2.1147	-5.1097
			XP_009145757.1	4.0878	4.7926	-1.3367	-3.0783
		MAM1	XP_009137065.1	2.0785	3.171	0	0
			XP_013672919.1	2.0785	0	0	0
			XP_009144531.1	0	0	-1.5188	-3.6644
			XP_009130133.1	3.7587	3.9308	-1.4385	-3.9348
			XP_009136397.1	1.6635	0	-0.6755	-2.6891
			XP_009136398.1	2.5786	2.9192	-1.1692	-1.1748
		IPMI- LSU1	XP_009107732.1	0.9747	1.1711	-0.5451	-1.0562
		IPMI-SSU2	XP_009142910.1	1.884	2.3539	-1.2788	-3.5244
		IPMDH1	XP_009125966.1	1.6831	2.0661	-1.8336	-3.7102
	Core structure synthesis	CYP79F1	XP_009149073.1	2.1117	1.8323	-2.0178	-4.7225
		SUR1	XP_009129641.1	0	0	-3.8145	-6.8425
		UGT74C1	XP_009141182.1	-0.7242	-1.4004	0	0
		SOT18	XP_009144531.1	0	0	-1.5842	-2.4967
			XP_009113467.1	0	0	-0.9993	-2.4967
			XP_013733135.1	0	0	-0.8473	-1.9118
			XP_009113161.1	0	0	-1.6773	-2.0373
	Side chain modification	FMO GS-OX2	XP_009113068.1	2.4384	1.756	0	0
		AOP1	XP_009122199.1	0	0	0.8751	1.0881
		AOP3	XP_009134376.1	1.5347	1.6616	-2.215	-3.3813
			XP_009144527.1	6.2485	6.756	0	0
Indolic pathway	Core structure synthesis	CYP79B2	ACR10253.1	1.2967	1.0482	-1.4642	-1.4902
			XP_009138546.1	1.1374	0.1711	-2.0879	-3.3258
			XP_013656565.1	1.1716	0	-1.1436	-0.7191
		CYP79B3	XP_009140345.1	2.0045	1.5496	-0.9665	-1.2908
		CYP83B1	XP_009107632.1	2.0969	1.4962	-0.8697	-1.2563
Transcription factor		IQD1-1	XP_009135043.1	0	0	-.21692	-2.0817
		MYB34	XP_009111962.1	0	0	-2.0773	-2.4967
Glucosinolate degradation (Myrosinase)		TGG4	XP_013726841.1	0	0	-0.8933	-4.4561
			XP_013654629.1	0	0	-1.4238	-2.9992
		TGG5	XP_013726832.1	0	0	-0.5008	-5.3041

Figure S6. The glucosinolate metabolic pathway and its expression pattern in leaves and roots exposed to drought stress. The absence genes listed in Supplementary Table 2 are marked in red. BCAT, Branched-chain amino acid aminotransferase; MAMI, Methylthioalkylmalate synthase; IPMI-LSU1, Isopropylmalate Isomerase Large Subunit 1; IPMI-SSU, Isopropylmalate Isomerase Small Subunit; IPMDH, Isopropylmalate Dehydrogenase; CYP, Cytochromes P450; GSTF, Glutathione S-transferases; GGP, Gamma-Glutamyl Peptidase; SUR, C-S lyase SUPERROOT; UGT, UDP-glucosyltransferase; SOT, Desulfoglucosinolate Sulfotransferases; FMO GS-OX, Flavin-Monooxygenase Glucosinolate S-Oxygenase; AOP, Alkenyl hydroxalkyl producing; GS-OH, Fe (II)-dependent oxygenase superfamily protein; BZO, Benzoyloxy glucosinolate; TGG, Myrosinase thioglucosidase; TSB1, Tryptophan synthase beta 1; IGMT, Indole glucosinolate methyl transferase; PEN2, Penetration 2.

Table S1. Gene ontology classification of differentially expressed genes (DEGs).

GO biology process	L1 vs.L2		L1 vs.L3		L2 vs.L3		R1 vs.R2		R1 vs. R2		R2 vs.R3	
	Up-regulated DEGs	Down-regulated DEGs	Up-regulated DEGs	Down-regulated DEGs	Up-regulated DEGs	Down-regulated DEGs	Up-regulated DEGs	Down-regulated DEGs	Up-regulated DEGs	Down-regulated DEGs	Up-regulated DEGs	Down-regulated DEGs
Biological process												
behavior	40	43	52	59	32	29	29	51	40	69	18	34
biological adhesion	116	162	140	193	87	98	84	149	103	222	49	99
biological phase	12	14	12	12	6	4	5	12	5	10	8	2
biological regulation	820	1063	1092	1251	640	654	600	1083	826	1585	463	722
cell aggregation	1	4	3	15	0	2	0	5	0	7	0	5
cell killing	21	26	28	34	17	13	11	25	17	39	10	21
cellular component organization or biogenesis	441	523	510	601	272	326	313	569	436	804	234	348
cellular process	1775	2139	2221	2588	1249	1375	1299	2353	1731	3330	1018	1523
detoxification	8	5	8	11	1	0	5	9	8	11	3	1
developmental process	348	443	450	547	242	279	271	435	362	691	184	291
growth	65	74	79	91	43	46	37	94	50	135	33	67
immune system process	210	270	257	335	144	185	140	254	181	401	112	169

localization	719	884	886	1015	507	556	548	940	724	1326	379	629
locomotion	110	171	136	194	70	88	94	179	125	262	59	112
metabolic process	1715	2000	2127	2416	1213	1288	1215	2258	1626	3202	951	1449
multicellular organismal process	418	514	527	618	303	328	305	539	424	841	216	361
multi-organism process	296	378	358	479	197	241	210	398	275	524	175	215
negative regulation of biological process	287	366	366	440	193	229	188	438	259	632	174	264
positive regulation of biological process	364	409	448	513	254	284	269	481	358	691	206	299
presynaptic process involved in chemical synaptic transmission	0	9	1	4	4	8	3	7	4	12	3	2
regulation of biological process	771	984	1016	1160	584	605	561	1007	772	1480	434	662
reproduction	86	108	102	111	52	54	68	95	89	143	41	61
reproductive process	84	107	99	110	52	54	66	94	88	140	41	58
response to stimulus	775	950	928	1122	545	616	550	924	750	1388	396	631

rhythmic process	14	10	14	18	7	6	11	8	18	13	5	9
signaling	419	463	513	567	299	327	303	516	411	773	203	361
single-organism process	1483	1718	1846	2068	1037	1127	1033	1916	1380	2765	799	1258
molecular function												
antioxidant activity	13	14	12	24	2	7	11	17	14	24	7	3
binding	1621	1957	2010	2307	1152	1224	1156	2125	1550	3034	864	1397
catalytic activity	1372	1658	1720	2032	971	1067	975	1896	1293	2653	797	1220
chemoattractant activity	4	3	6	6	0	0	2	3	2	10	0	3
electron carrier activity	37	61	46	97	25	44	30	52	40	86	14	43
molecular function regulator	67	107	88	113	53	52	45	89	55	134	28	53
molecular transducer activity	92	124	113	148	54	81	56	107	83	162	41	89
nucleic acid binding transcription factor activity	80	113	111	141	71	59	65	105	91	167	46	73
nutrient reservoir activity	1	3	1	6	1	1	0	1	0	3	0	1

signal transducer activity	120	127	137	159	72	99	69	128	99	186	53	98
structural molecule activity	132	173	146	196	92	100	125	162	159	236	64	116
transcription factor activity, protein binding	27	38	51	49	26	32	34	37	42	51	15	24
translation regulator activity	9	2	8	5	3	3	6	8	6	11	7	4
transporter activity	240	314	285	376	160	197	170	283	219	411	125	182
cellular component												
cell	1477	1728	1848	2088	1036	1112	1020	1909	1394	2694	816	1247
cell junction	53	77	51	98	32	46	48	72	53	129	20	61
cell part	1477	1728	1848	2088	1036	1112	1020	1909	1394	2694	816	1247
extracellular region	350	376	415	498	243	275	237	447	330	658	175	303
extracellular region part	236	269	295	333	169	186	159	298	224	462	116	205
macromolecular complex	726	839	852	996	496	518	509	840	678	1212	0	554
membrane	986	1187	1181	1411	689	760	697	1248	937	1823	529	831
membrane part	514	689	610	802	381	403	358	674	500	998	251	470

membrane-enclosed lumen	156	199	212	251	109	136	109	224	150	319	76	127
nucleoid	7	12	11	7	4	0	9	9	11	8	1	3
organelle	820	958	1026	1160	562	624	590	1065	811	1534	447	685
organelle part	459	544	568	661	313	358	321	604	430	888	237	389
other organism	88	102	79	149	46	81	54	131	77	161	68	62
other organism part	88	102	79	149	46	81	54	131	77	161	68	62
supramolecular complex	52	69	69	71	44	29	44	59	50	97	35	42
synapse	36	62	54	73	35	38	36	64	39	95	16	49
synapse part	29	52	39	56	24	27	27	47	26	74	10	34
virion	85	102	106	147	57	67	76	114	99	155	68	55
virion part	64	76	81	101	40	48	57	91	80	109	54	34

Table S2. Differentially expressed genes involved in S-misc BIN.

BinCode	BinName	At Id	Gene description	Gene bank Id
16.5.1	secondary metabolism. sulfur-containing. glucosinolates	At4g03070	probable 2-oxoglutarate-dependent dioxygenase AOP1	XP_009122199.1
16.5.1.1.1.1	secondary metabolism. sulfur-containing. glucosinolates. synthesis. aliphatic. branched-chain amino acid aminotransferase (BCAT/MAAT)	At3g19710	methionine aminotransferase BCAT4-like	XP_009135648.1
16.5.1.1.1.1	secondary metabolism. sulfur-containing. glucosinolates. synthesis. aliphatic. branched-chain amino acid aminotransferase (BCAT/MAAT)	At3g19710	methionine aminotransferase BCAT4	XP_009145757.1
16.5.1.1.1.2	secondary metabolism. sulfur-containing. glucosinolates. synthesis. aliphatic. methylthioalkylmalate synthase (MAMI)	At5g23010	methylthioalkylmalate synthase 2, chloroplastic-like, partial	XP_009137065.1
16.5.1.1.1.2	secondary metabolism. sulfur-containing. glucosinolates. synthesis. aliphatic. methylthioalkylmalate synthase (MAMI)	At5g23010	methylthioalkylmalate synthase 2, chloroplastic-like isoform X1	XP_013672919.1
16.5.1.1.1.2	secondary metabolism. sulfur-containing. glucosinolates. synthesis. aliphatic. methylthioalkylmalate synthase (MAMI)	At5g23010	methylthioalkylmalate synthase 1, chloroplastic-like	XP_009144531.1
16.5.1.1.1.2	secondary metabolism. sulfur-containing. glucosinolates. synthesis. aliphatic. methylthioalkylmalate synthase (MAMI)	At5g23010	methylthioalkylmalate synthase 1, chloroplastic-like	XP_009130133.1
16.5.1.1.1.2	secondary metabolism. sulfur-containing. glucosinolates. synthesis. aliphatic. methylthioalkylmalate synthase (MAMI)	At5g23010	methylthioalkylmalate synthase 1, chloroplastic-like	XP_009136397.1
16.5.1.1.1.2	secondary metabolism. sulfur-containing. glucosinolates. synthesis. aliphatic. methylthioalkylmalate synthase (MAMI)	At5g23010	methylthioalkylmalate synthase 1, chloroplastic-like isoform X1	XP_009136398.1
16.5.1.1.1.3	secondary metabolism. sulfur-containing. glucosinolates. synthesis. aliphatic. methylthioalkylmalate isomerase large subunit (MAMI-IL)	At4g13430	3-isopropylmalate dehydratase large subunit isoform X1	XP_009107732.1
16.5.1.1.1.4	secondary metabolism. sulfur-containing. glucosinolates. synthesis. aliphatic. methylthioalkylmalate isomerase small subunit (MAMI-IS)	At2g43100	3-isopropylmalate dehydratase small subunit 1	XP_009142910.1
16.5.1.1.1.5	secondary metabolism. sulfur-containing. glucosinolates. synthesis. aliphatic. methylthioalkylmalate dehydrogenase (MAMI-D)	At5g14200	3-isopropylmalate dehydrogenase 3, chloroplastic	XP_009125966.1

16.5.1.1.1.6	secondary metabolism. sulfur-containing. glucosinolates. synthesis. aliphatic. CYP79F1/F2 monooxygenase	At1g16410	dihomomethionine N-hydroxylase-like	XP_009149073.1
16.5.1.1.1.8	secondary metabolism. sulfur-containing. glucosinolates. synthesis. aliphatic. UDP-glycosyltransferase	At2g31790	UDP-glycosyltransferase 74C1-like	XP_009141182.1
16.5.1.1.1.9	secondary metabolism. sulfur-containing. glucosinolates. synthesis. aliphatic. sulfotransferase	At1g74090	cytosolic sulfotransferase 18-like	XP_009106068.1
16.5.1.1.1.9	secondary metabolism. sulfur-containing. glucosinolates. synthesis. aliphatic. sulfotransferase	At1g74090	cytosolic sulfotransferase 18-like	XP_009113467.1
16.5.1.1.1.9	secondary metabolism. sulfur-containing. glucosinolates. synthesis. aliphatic. sulfotransferase	At1g74090	cytosolic sulfotransferase 18-like	XP_013733135.1
16.5.1.1.1.9	secondary metabolism. sulfur-containing. glucosinolates. synthesis. aliphatic. sulfotransferase	At1g74090	cytosolic sulfotransferase 18-like	XP_009113161.1
16.5.1.1.1.10	secondary metabolism. sulfur-containing. glucosinolates. synthesis. aliphatic. flavin-containing monooxygenase	At1g62540	flavin-containing monooxygenase FMO GS-OX2-like	XP_009113068.1
16.5.1.1.1.11	secondary metabolism. sulfur-containing. glucosinolates. synthesis. aliphatic. glucosinolate 2-oxoglutarate-dependent dioxygenase (AOP)	At4g03050	probable 2-oxoglutarate-dependent dioxygenase AOP1 isoform X1	XP_009144527.1
16.5.1.1.1.11	secondary metabolism. sulfur-containing. glucosinolates. synthesis. aliphatic. glucosinolate 2-oxoglutarate-dependent dioxygenase (AOP)	At4g03050	probable 2-oxoglutarate-dependent dioxygenase AOP1	XP_009134376.1
16.5.1.1.1.12	secondary metabolism. sulfur-containing. glucosinolates. synthesis. aliphatic. benzoate-CoA ligase	At1g65890	probable acyl-activating enzyme 12, peroxisomal isoform X1	XP_009124154.1
16.5.1.1.3.1	secondary metabolism. sulfur-containing. glucosinolates. synthesis. indole. CYP79B2 monooxygenase	At4g39950	cytochrome P450 79b2	ACR10253.1
16.5.1.1.3.1	secondary metabolism. sulfur-containing. glucosinolates. synthesis. indole. CYP79B2 monooxygenase	At4g39950	cytochrome P450 79B1-like	XP_009138546.1
16.5.1.1.3.1	secondary metabolism. sulfur-containing. glucosinolates. synthesis. indole. CYP79B2 monooxygenase	At2g22330	tryptophan N-monooxygenase 2	XP_009140345.1
16.5.1.1.3.1	secondary metabolism. sulfur-containing. glucosinolates. synthesis. indole. CYP79B2 monooxygenase	At4g39950	cytochrome P450 79B1-like	XP_013656565.1

16.5.1.1.4.1	secondary metabolism. sulfur-containing. glucosinolates. synthesis. shared. CYP83B1 phenylacetaldoxime monooxygenase	At4g31500	cytochrome P450 83B1	XP_009107632.1
16.5.1.1.4.2	secondary metabolism. sulfur-containing. glucosinolates. synthesis. shared. alkylthiohydroximate C-S lyase	At4g28420	probable aminotransferase TAT1	XP_009129641.1
16.5.1.2.3	secondary metabolism. sulfur-containing. glucosinolates. regulation. indole	At3g09710	protein IQ-DOMAIN 1-like	XP_009135043.1
16.5.1.2.3	secondary metabolism. sulfur-containing. glucosinolates. regulation. indole	At5g60890	transcription factor MYB34-like	XP_009111962.1
16.5.1.3.1	secondary metabolism. sulfur-containing. glucosinolates. degradation.myrosinase	At1g54020	GDSL esterase/lipase At1g54020-like	XP_009147560.1
16.5.1.3.1	secondary metabolism. sulfur-containing. glucosinolates. degradation. myrosinase	At1g47600	myrosinase 4-like	XP_013726841.1
16.5.1.3.1	secondary metabolism. sulfur-containing. glucosinolates. degradation. myrosinase	At1g51470	myrosinase 4-like	XP_013726832.1
16.5.1.3.1	secondary metabolism. sulfur-containing. glucosinolates. degradation. myrosinase	At1g47600	myrosinase 4	XP_013654629.1
16.5.1.3.1	secondary metabolism. sulfur-containing. glucosinolates. degradation. myrosinase	At1g54010	inactive GDSL esterase/lipase-like protein 23	XP_009107056.1
16.5.1.3.2	secondary metabolism. sulfur-containing. glucosinolates. degradation .nitrilespecifier protein	At5g48180	nitrile-specifier protein 5-like	XP_009129713.1
16.5.1.3.2	secondary metabolism. sulfur-containing. glucosinolates. degradation. nitrilespecifier protein	At3g16400	LOW QUALITY PROTEIN: nitrile-specifier protein 1-like	XP_009146087.1
16.5.1.3.2.1	secondary metabolism. sulfur-containing. glucosinolates. degradation. nitrilespecifier protein. epithiospecifier protein	At1g54040	epithiospecifier protein-like	XP_009147556.1
16.5.1.3.2.1	secondary metabolism. sulfur-containing. glucosinolates. degradation. nitrilespecifier protein. epithiospecifier protein	At1g54040	epithiospecifier protein-like	XP_009147566.1

Table S3. Sequences of primers used for qPCR analysis.

Gene	Forward primer	Reverse primer
XP_009111076.1	TGGCCGTGGAAGCAGAGAAC	GGACCAGTCAGAGGGTTCGG
XP_009112062.1	GGTTCCTTACGTGCTTAACC	CCATCATCTCAGCCTGTTTC
XP_009101738.1	AGTGATGATGGAGCCTTTGG	CAACTTGGTAAGCGTGCTTC
XP_009138305.1	TGGACTCGTCGTCGTCTGGA	TCTCCTCGCTGATTCACGGT
XP_009111876.1	CGCGGGTTTGAGATCGGAGA	ATCCGCGTTTGCCTCCTGTT
XP_009123528.1	AGTGTCAGCAGATGGGATAG	CTTCTTTGCCTCCTCTCAAC
XP_009135624.1	AACAACGGTGCTGCTGAAGG	TGATGCTGCTGCTGTGGATG
XP_009145807.1	GGTGGGTATGGTCAACAAG	TAAGGAACTGGCGACAGAG
XP_009104023.1	ACACAGCCTCGGTCATTCTTTC	TCCTTCCTGGAGTTTGCGTATC
XP_009107646.1	TGGACCAGCAATCGGTGTCG	CTCCTTCCCGGCATCATCGG
XP_009117601.1	TGGTGTGGTTAGGGAAGAG	CGGCTGAGTTTGAGTTGTC
XP_009146627.1	TTCTTGTGGCTTGGCGGGTT	TCTTCAGCCTGCTGGGAGGT
XP_009106517.1	CGCACGCTCACACTTGCTTC	ACCGTTCTTGCCACAAGACTCA
XP_009133819.1	GCCGTTCCCAGCCCTTGTTA	GCCCTGTGAACTGACGGTGT
Actin	TGGGCGTACTACTGGTATTGTG	TGTACCCTCTCTCGGTGAGAAT
